# The effect of structured hand massage on hand function and grip strength in rheumatoid arthritis patients: a randomized controlled pilot study

**DOI:** 10.1007/s00296-026-06095-3

**Published:** 2026-04-06

**Authors:** Umida Khojakulova, Gülşah Yaşa Öztürk, Burak Okyar, Olena Zimba, Burhan Fatih Kocyigit

**Affiliations:** 1 Department of Emergency Medicine and Nursing, South Kazakhstan Medical Academy, Shymkent, Kazakhstan; 2 Faculty of Medicine, Department of Physical Medicine and Rehabilitation, University of Health Sciences, Adana City Training and Research Hospital, Adana, Türkiye; 3 Department of Rheumatology, Adana City Training and Research Hospital, Adana, Türkiye; 4 Department of Rheumatology, Immunology and Internal Medicine, University Hospital in Kraków, Kraków, Poland; 5 National Institute of Geriatrics, Rheumatology and Rehabilitation, Warsaw, Poland; 6 Department of Internal Medicine N2, Danylo Halytsky Lviv National Medical University, Lviv, Ukraine

**Keywords:** Rheumatoid Arthritis, Massage Therapy, Musculoskeletal Manipulations, Hand, Hand Grip Strength, Pinch Strength

## Abstract

**Introduction:**

This study aimed to assess the impact of a 10-session hand massage protocol on hand function, grip strength, pinch strength, pain threshold, and tactile sensory threshold in patients with early-stage, seropositive rheumatoid arthritis (RA) without radiographic damage or hand deformity.

**Methods:**

This study was a randomized controlled clinical trial. Thirty female RA patients with a diagnosis time of less than two years, seropositive, with low disease activity/remission, and without active arthritis in their hands were included in the study. Participants were randomized 1:1 to intervention and control groups. The intervention group underwent 10 hand massage sessions over a two-week period, in addition to regular medical care. The control group received only regular medical care. All assessments were performed at baseline (T1) and at week 2 (T2). Outcome measures included hand grip strength, pinch strength, pain threshold (dolorimetry), tactile sensory threshold (Semmes-Weinstein monofilament test), Duruöz Hand Index (DHI), and patient global assessment (PGA).

**Results:**

A total of 30 RA patients were included in the study (intervention group, *n* = 15; control group, *n* = 15). At baseline, no significant differences were observed between the groups in demographic, laboratory, or clinical characteristics (*p* > 0.05). In the intervention group, significant improvements in hand grip strength and pinch force were observed at T2 compared with baseline (*p* = 0.008 and *p* = 0.023, respectively). Median hand grip strength increased from 55 (40–75) to 65 (45–80) kg, and median pinch strength increased from 15 (6–18) to 16 (11–21) kg. No significant alterations were noted in these parameters within the control group (*p* > 0.05). In the control group, median hand grip strength was 45 (25–80) kg at baseline and 45 (20–80) kg at week 2, while median pinch strength was 14 (6–18) kg at baseline and 14 (5–19) kg at week 2. In the intervention group, significant reductions in DHI and PGA scores were recorded at the end of therapy (*p* = 0.005 and *p* = 0.001, respectively). No significant differences were noted in DHI and PGA levels in the control group (*p* > 0.05). Moreover, median PGA scores were significantly lower in the intervention group than in the control group at T2 (4 [2–5] vs. 5 [3–6]; *p* < 0.001). No significant changes were found in dolorimeter and touch sensory thresholds in either group (*p* > 0.05).

**Conclusion:**

Hand massage interventions for patients with RA enhance grip strength, hand function, and overall patient evaluations. No effect on pain or tactile thresholds was observed. Further studies with larger sample sizes and longer follow-up periods are required to corroborate these findings.

**Supplementary Information:**

The online version contains supplementary material available at 10.1007/s00296-026-06095-3.

## Introduction

Rheumatoid arthritis (RA) is a chronic autoimmune inflammatory disease that primarily impacts synovial joints, resulting in pain, swelling, morning stiffness, and gradual joint deterioration [1]. Frequent engagement of the hand and wrist joints during the disease course lead to diminished grip strength, compromised fine motor skills, and restrictions in daily activities [2, 3]. The impairment of hand function diminishes autonomy and adversely affects quality of life of RA patients [4].

The duration of the disease and the level of joint destruction in RA significantly influence the emergence of functional impairments. In the early stages, implementing appropriate medical interventions and rehabilitation strategies is essential for managing inflammation and preventing joint disorders [5, 6]. In patients without radiological damage or hand abnormalities, maintaining hand function is crucial to avert irreversible functional impairments.

The primary strategy in RA management is to control disease activity and prevent structural damage. Due to the complex clinical ramifications of RA, rehabilitation strategies are essential for pain alleviation, maintaining independence, and enhancing quality of life [7]. Hand massage offers a supportive approach for patients with RA, facilitating soft-tissue relaxation, enhancing local circulation, and alleviating muscular spasms [8]. Moreover, massage therapy may facilitate pain regulation by mechanical stimulation, diminish sensitivity, and enhance functional mobility by improving the flexibility of the tissues surrounding the joints. Hand massage is particularly beneficial for fine motor skills and grip strength [9].

This study aims to investigate the impact of 10 sessions of hand massage, administered in conjunction with regular medical treatment, on hand function, grip strength, pinch force, pain threshold, and sensory parameters in seropositive RA patients diagnosed within two years, without radiological damage or deformity in the hands, in comparison to a group receiving routine rheumatic care. The study also aims to detect differences within and between groups by assessing pre- and post-treatment changes in both groups.

## Methods

### Study design and participants

This study was designed as a randomized controlled pilot clinical trial. The study was conducted between October 1, 2025 and January 31, 2026. Participants aged 18 and older with RA who met the 2010 ACR/EULAR classification criteria were recruited [10]. All participants were clinically stable and maintained their regular rheumatological follow-up and routine medical treatment without interruption during the trial period.

The inclusion criteria were the availability of complete clinical and laboratory records, consistent follow-up, seropositivity, diagnosis date within the preceding two years, and documented informed permission for research participation. None of the patients had hand deformities or any radiological damage. Conventional plain radiography (direct X-ray) of the hands was employed to confirm the absence of erosions or radiographic damage. The exclusion criteria encompassed concurrent systemic autoimmune disorders, malignancies, active infections, pregnancy, severe cervical radiculopathy, previous upper extremity fracture, brachial plexopathy, and a prior diagnosis of neurological condition potentially impacting upper extremity performance. Only patients with low disease activity or in remission were included in the study. Patients exhibiting active arthritis in the hand and wrist joints were excluded.

### Randomization

Participants were assigned to two groups in a 1:1 ratio using computer-assisted randomization. The control group was on standard rheumatological follow-up and routine medical treatment without any additional interventions. The intervention group of patients, in conjunction with their regular rheumatological follow-up and medical treatment, participated in a standardized hand massage program designed to enhance hand function.

All participants underwent evaluation at two time points: the baseline assessment prior to the intervention (T1; week 0) and at week 2 following completion of the intervention (T2). This study did not employ blinding.

### Structured massage therapy program

A structured hand massage procedure was administered to the intervention group of patients with RA. The massage therapy included five sessions per week, totaling 10 sessions over two weeks. Each session lasted around 40 min, and the applications were conducted in a quiet, comfortable setting with an appropriate temperature. The key goals of the protocol included preserving range of motion, reducing edema and stiffness, alleviating muscle spasms and pain, and enhancing hand function to facilitate daily activities. The massage procedure consisted of four stages. In the first stage, gentle effleurage was applied to the dorsal and palmar surfaces of the hand for approximately 5 min to stimulate superficial circulation, followed by light stroking in a lymphatic drainage technique from the fingers proximally to manage edema. During the second stage, gentle circular friction was applied to the intrinsic muscles of the palmar and dorsal aspects for approximately 10 min to promote relaxation, accompanied by mild petrissage of the thenar and hypothenar muscle groups. In the third stage, joint-focused massage was performed for approximately 20 min: low-pressure circular massage was applied around the hand joints, and soft-tissue mobilization was supported by longitudinal effleurage between the joints. At this juncture, passive mobilization strategies were employed without undue force, in accordance with range-of-motion assessments. The fingers were softly stroked one by one, from proximal to distal, with flexion-extension motions and gentle stretching. Additionally, moderate rubbing and effleurage were used around the wrist to enhance local circulation and promote relaxation. The procedure was completed with superficial effleurage administered to the entire hand for about 5 min [11, 12]. All massage sessions were conducted with strict adherence to the standardized structured massage therapy protocol to ensure consistency throughout the intervention period.

### Demographic and clinical data

Demographic and clinical characteristics were documented at the start of the trial. All participants were female. Demographic information, including age, body mass index (BMI), disease duration, dominant hand, and smoking and alcohol use, was recorded. Laboratory assessments included measurements of rheumatoid factor (RF), anti-cyclic citrullinated peptide antibody (Anti-CCP), erythrocyte sedimentation rate (ESR), and C-reactive protein (CRP) concentrations. Disease activity was assessed using the Disease Activity Score based on 28 joints using C-reactive protein (DAS28-CRP), Simplified Disease Activity Index (SDAI), and Clinical Disease Activity Index (CDAI).

### DAS28-CRP

The DAS28-CRP index was calculated from the counts of tender and swollen joints, serum CRP concentration, and a comprehensive assessment of the patient’s overall health status across 28 joints [13]. To ensure the safe implementation and tolerability of massage therapy for participants, the study included only patients in remission or with low disease activity, as defined by the DAS28-CRP score; those with moderate or high disease activity were excluded.

### SDAI

The SDAI calculation involved assessing the number of tender and swollen joints from 28 joints, whereas the patient’s and physician’s global assessments were documented using a 0–10 cm visual analog scale (VAS). Serum CRP levels were quantified and used in the computation. The SDAI score was derived by summing the scores for these criteria [14].

### CDAI

The number of tender and swollen joints was counted across 28 joints, and the patient’s and physician’s global assessments were measured using a 0–10 cm VAS. The CDAI score is computed by summing these clinical parameters and is used to evaluate disease activity regardless of laboratory results [15].

### Outcome assessments

#### Hand grip strength

Grip strength was evaluated with a Jamar hydraulic hand dynamometer (Baseline Hydraulic Hand Dynamometer, Irvington, NY, USA). Participants were evaluated while seated with back support, shoulder adduction, and neutral rotation. The elbow was flexed at 90°, the forearm was in a neutral position, and the wrist was positioned at around 0–30° of extension. Measurements were conducted on the dominant hand, and participants were instructed to exert maximal effort on the dynamometer. Each measurement was performed three times with brief rest periods, and the maximum value was recorded for analysis [16]. Measurement results were recorded in kilograms (kg). In addition to baseline (T1) and post-treatment (T2) assessments, grip strength was also recorded at each massage session in the intervention group to evaluate daily progression over the 10-day intervention period.

#### Pinchmeter

Pinch strength was evaluated with a pinch gauge (Baseline Hydraulic Pinch Gauge, Irvington, NY, USA). Participants were evaluated while sitting with back support during the measures. Participants were instructed to apply maximal force with the pinch gauge between the thumb and index finger (palmar pinch strength), and the value was recorded. Each measurement was performed three times, with brief rest periods between to mitigate fatigue. The maximum recorded value was utilized for analysis [17]. Measurement results were recorded in kg.

### Pain threshold

A manual dolorimeter (Fabrication Enterprises Baseline Dolorimeter, USA) was used to assess subjects’ pain thresholds. The dolorimeter is a measuring device comprising a metal tip and a hand grip, designed to exert pressure over a fixed contact area of 1 cm² and calibrated to indicate the applied pressure in kg/cm². Participants were sitting during the examination, and measurements were obtained from the dorsal surface of the dominant wrist. For each participant, three consecutive measures were obtained, and the mean of these measurements was documented for analysis. Throughout the application, pressure was increased incrementally, and participants were instructed to respond with “stop” upon experiencing discomfort. The pressure value displayed on the device at the time of the warning was used as the pain threshold. Measurements were documented in kg/cm² [18].

### Tactile sensory threshold

The Semmes-Weinstein Monofilament Test (SWMT) (North Coast Medical, Inc., Morgan Hill, CA, USA) was used to assess the tactile sensory threshold by measuring superficial sensation on the dorsum of the hand. This assessment is a standardized threshold-measuring technique that objectively assesses light-touch sensation and identifies the minimal mechanical stimulus an individual can recognize. The evaluation was conducted using monofilaments of varying thickness and elasticity. Each monofilament is engineered to produce a specific force upon application and has distinct values arranged on a logarithmic scale. These values represent the gram-equivalent pressure exerted on the skin, providing quantitative data on peripheral sensory function. During application, the monofilament was positioned perpendicular to the skin surface and applied pressure for roughly one second to form a recognizable “C” shape. The participant’s perception of the thinnest monofilament was recorded as the tactile sensory threshold for that specific location [19].

The Duruöz Hand Index (DHI) was used to assess hand function and limitations in daily activities. The DHI is a validated and reliable assessment instrument designed to evaluate the degree of hand impairment in rheumatic conditions. The scale comprises 18 items assessing everyday tasks that require manual skills. These questions assess physical ability for self-care activities, domestic tasks, occupational duties, clothing, and overall motor skills. Participants were instructed to evaluate each task on a scale from 0 (no difficulty) to 5 (unable/completely debilitated), reflecting their performance throughout the preceding week. The overall score ranged from 0 to 90, with higher scores indicating greater impairment in hand function and functional restrictions [20, 21].

### Patient global assessment

The Patient Global Assessment (PGA) was used to evaluate patients’ perceptions of their overall disease state. Participants rated disease severity on a 0–10 scale, and the scores were recorded [22].

**Ethics statement**

The study received ethics approval from the Ethics Committee of Adana City Training and Research Hospital on September 25, 2025 (decision number 713). The ethics approval was also obtained from the Local Bioethics Committee of South Kazakhstan Medical Academy on January 31, 2023 (protocol N1). Written informed consent was obtained from all participants.

### Statistical Analysis

Statistical analyses were conducted using IBM SPSS Statistics for Windows, Version 20.0 (IBM Corp., Armonk, NY, USA). Normality of the data distribution was assessed using the Shapiro-Wilk test. Categorical variables were reported as numbers (n) and percentages (%), whereas continuous variables were expressed as medians (minimum - maximum). For intergroup comparisons, the Mann-Whitney U test was used for continuous data, and the Chi-square test for categorical variables. The Wilcoxon Signed-Rank test was used to compare within-group changes (T1-T2). Trend analysis was performed to evaluate changes in handgrip strength across sessions in the intervention group, and linear regression was used to quantify these changes. All analyses were considered statistically significant at *p* < 0.05.

## Results

A total of 30 RA patients were included in the study: 15 patients were assigned to the intervention group and 15 to the control group. The median age was 54 (31–70) years in the intervention group and 55 (22–71) years in the control group. The median BMI was 27.3 kg/m² (21.3–37.1) in the intervention group and 27.7 kg/m² (19.1–35.3) in the control group. There was no statistically significant difference between groups in terms of age, BMI, disease duration, dominant hand distribution, smoking, and alcohol use (*p* > 0.05) (Table 1).

**Table 1:** Baseline characteristics of the groups

	Intervention group (*n* = 15)	Control group (*n* = 15)	*p*
Age (year)	54 (31–70)	55 (22–71)	0.663*
BMI (kg/m^2^)	27.3 (21.3–37.1)	27.7 (19.1–35.3)	0.740*
Disease duration (month)	12 (4–23)	17 (4–23)	0.417*
Dominant hand (n,%) Right Left	12 (80) 3 (20)	9 (60) 6 (40)	0.232**
Smoking status (n,%) Yes No	3 (20) 12 (80)	5 (33.3) 10 (66.7)	0.409**
Alcohol consumption status (n,%) Yes No	0 (0) 15 (100)	0 (0) 15 (100)	-

* Mann-Whitney U test ** Chi-Square test

*n*: number; %: percentage; *BMI*: Body mass index

No statistically significant differences were detected between the groups in terms of RF, anti-CCP, ESR, CRP, DAS28-CRP, SDAI, and CDAI values ​​(*p* > 0.05). Disease activity values ​​and laboratory parameters of the intervention and control groups are presented in Table 2.

**Table 2:** Comparison of intervention and control groups in terms of clinical and laboratory parameters

Parameter	Intervention group (*n* = 15)	Control group (*n* = 15)	*p*
RF (IU/mL)	50 (23–150)	35 (23–178)	0.547^*^
Anti-CCP (U/mL)	190 (32–500)	96 (23–200)	0.067^*^
ESR(mm/h)	13 (2–29)	15 (6–21)	0.967^*^
CRP(mg/L)	3.8 (1.3–8.5)	4.1 (0.9–7.3)	0.803^*^
DAS28-CRP	1.88 (0.56–2.43)	1.98 (1.30–2.19)	0.934^*^
SDAI	9.1 (6.4–11.3)	8.4 (4.1–10.7)	0.340^*^
CDAI	8 (6–11)	8 (4–10)	0.447^*^

* Mann-Whitney U test

*n*: number; *RF*: Rheumatoid Factor; *Anti-CCP*: Anti–Cyclic Citrullinated Peptide Antibody; *ESR*: Erythrocyte Sedimentation Rate; *CRP*: C-Reactive Protein; *DAS28-CRP*: Disease Activity Score based on 28 joints using C-reactive protein; *SDAI*: Simplified Disease Activity Index; *CDAI*: Clinical Disease Activity Index; *N*: Normal Range

Table 3 presents intra-group and inter-group comparisons of dominant extremity handgrip strength and pinchmeter measurements. At baseline (T1), no statistically significant difference was observed between the groups in terms of handgrip strength and pinchmeter measurements (*p* > 0.05). In the intervention group, a statistically significant increase in handgrip strength was observed at the end of treatment (T2) compared with baseline (*p* = 0.008). In contrast, no statistically significant change was detected in handgrip strength values ​​in the control group (*p* = 0.595). When pinchmeter measurements were evaluated, a statistically significant increase was discovered in the intervention group at T2 compared to baseline (*p* = 0.023). In the control group, no significant change was found in pinchmeter measurements in the within-group comparison (*p* = 0.164). For pinchmeter measurements, there was no statistically significant difference between the groups at T2 (*p* = 0.517).

**Table 3:** Intergroup and intragroup comparisons of hand grip strength and pinchmeter assessments

		Intervention group (*n* = 15)	Control group (*n* = 15)	*p*
	median (minimum-maximum)		median (minimum-maximum)	
HGS-dominant extremity (T1)	55 (40–75)		45 (25–80)	0.274*
HGS-dominant extremity (T2)	65 (45–80)		45 (20–80)	0.004*
	p^1^ = 0.008**		p^1^ = 0.595**	
Pinchmeter-dominant extremity (T1)	15 (6–18)		14 (6–18)	0.644*
Pinchmeter-dominant extremity (T2)	16 (11–21)		14 (5–19)	0.517*
	p^1^ = 0.023**		p^1^ = 0.164**	

* Mann Whitney U test; **Wilcoxon Signed-Rank Test; HGS: Hand grip strength

T1 = baseline, T2 = at the end of the massage therapy program (T2 = week 2)

p^1^= baseline - T2 (week 2); n: number

Table 4 presents within- and between-group comparisons of dolorimetry, SWMT, DHI, and PGA measurements between the intervention and control groups. At baseline (T1), there were no statistically significant differences between the groups in dolorimeter, SWMT, DHI, and PGA values (*p* > 0.05). A statistically significant reduction in DHI scores was observed in the intervention group at the end of treatment (T2) in within-group comparisons (*p* = 0.005). In contrast, there was no significant difference in DHI scores in the control group (*p* = 0.4). No statistically significant difference was found between baseline and end-of-treatment measurements in within-group comparisons of dolorimeter and SWMT measurements (for both groups) (*p* > 0.05). No statistically significant differences were detected between the control and intervention groups in dolorimeter, SWMT, and DHI values at the end of treatment (T2) in intergroup comparisons (*p* > 0.05). In within-group comparisons, the intervention group showed a statistically significant decrease in PGA scores at the end of treatment (T2) compared with baseline (*p* = 0.001). In the control group, no statistically significant change in PGA scores was observed (*p* = 0.564). In between-group comparisons, the intervention group had significantly lower PGA scores than the control group at the end of treatment (*p* < 0.001).

**Table 4:** Intergroup and intragroup comparisons of pain threshold, tactile sensory threshold, DHI, and PGA assessments

		Intervention group (*n* = 15)	Control group (*n* = 15)	*p*
		median (minimum-maximum)	median (minimum-maximum)	
Dolorimeter-dominant extremity (T1)	12 (10–13)		12 (10–15)	0.791*
Dolorimeter-dominant extremity (T2)	12 (9–13)		11 (10–16)	0.879*
	p^1^ = 0.776**		p^1^ = 0.675**	
SWMT-dominant extremity (T1)	1.65 (0- 3.22)		1.65 (1.65–2.83)	0.201*
SWMT-dominant extremity (T2)	1.65 (0- 2.83)		1.65 (1.56–2.83)	0.263*
	p^1^ = 0.100**		p^1^ = 0.18**	
DHI (T1)	19 (0–72)		24 (0–90)	0.6*
DHI (T2)	9 (0–54)		25 (0–90)	0.144*
	p^1^ = 0.005**		p^1^ = 0.4**	
PGA (T1)	5 (4–7)		5 (3–7)	0.895*
PGA (T2)	4 (2–5)		5 (3–6)	< 0.001*
	p^1^ = 0.001**		p^1^ = 0.564**	

* Mann Whitney U test; **Wilcoxon Signed-Rank Test; HGS: Hand grip strength; T1 = baseline, T2 = at the end of the massage therapy program (T2 = week 2); p^1^= baseline - T2 (week 2); SWMT: Semmes-Weinstein Monofilament Test; DHI: Duruöz Hand Index; PGA: Patient Global Assessment; n: number

**Fig. 1:**
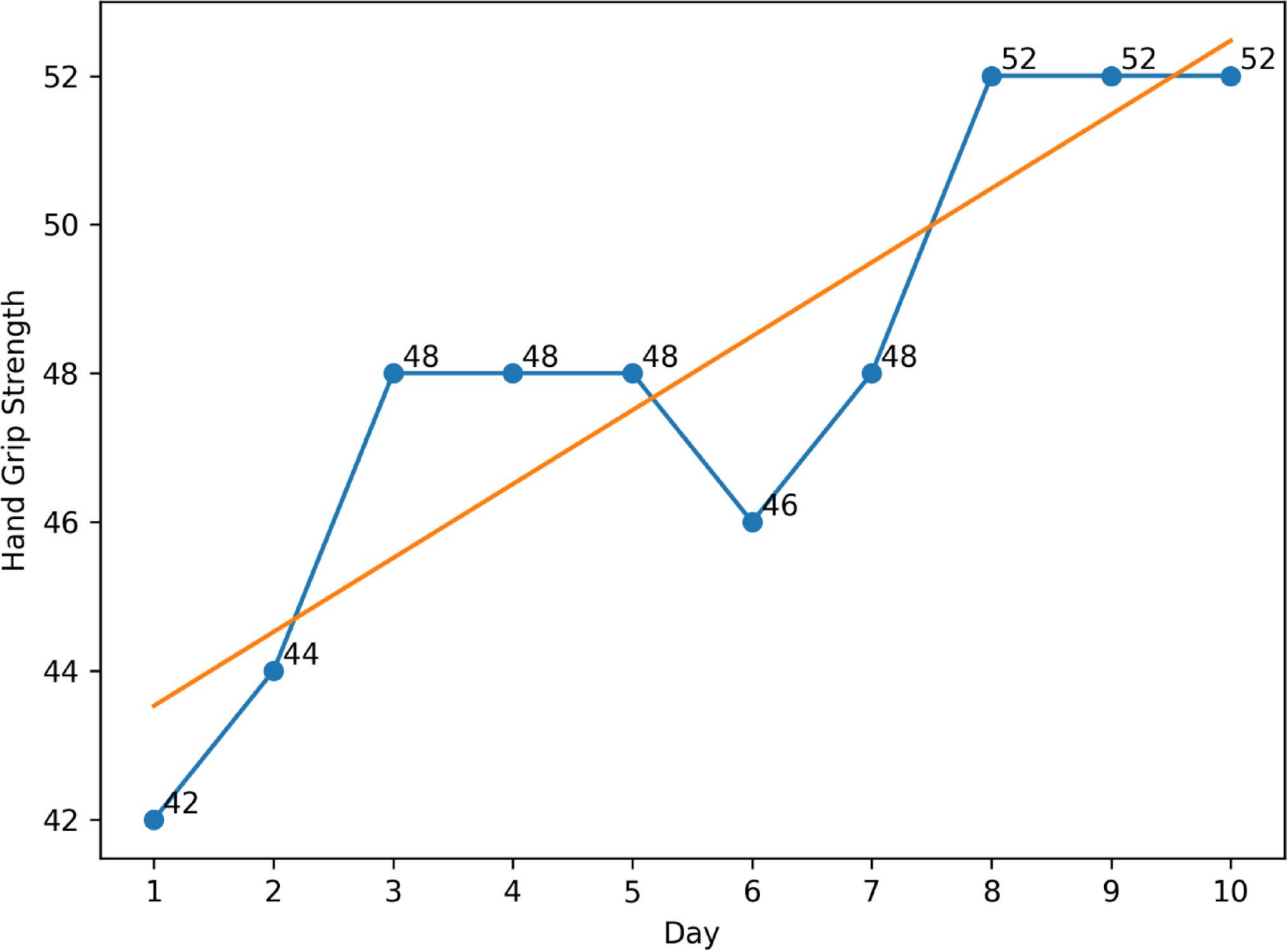
Ten-day trend analysis of handgrip strength in the intervention group. *Grip strength was measured at each of the 10 massage sessions using a hand-held dynamometer. The plotted values represent the median dominant-hand grip strength (kg) of the participants for each intervention day. The regression line reflects the overall trend across the 10-day intervention period*

The change in handgrip strength over the 10-day period in patients within the intervention group (*n* = 15) was analyzed using patient-specific linear regression. The results showed a significant increase in handgrip strength over time in 8 of the 15 patients (*p* < 0.05). For the remaining patients, the change was not statistically significant (*p* > 0.05). When data from all patients in the intervention group were combined, a group-level linear regression indicated a significant upward trend in handgrip strength (*p* = 0.007) (Fig. 1).

No flare-ups occurred during the intervention period; no adverse reactions were noted, and no participants withdrew from the follow-up program.

## Discussion

This study assessed the effect of a 10-session hand massage on hand function and clinical outcomes in seropositive RA patients diagnosed within the past 2 years and without radiographic hand damage. The results indicated a significant enhancement in handgrip strength and a substantial reduction in DHI scores at the end of therapy within the intervention group. A notable enhancement in PGA scores was observed in the intervention group. No significant alterations in pain and tactile sensory thresholds were observed in either group. Moreover, handgrip strength in the intervention group improved substantially across sessions, with a notable upward trend over time, assessed by linear regression.

The intervention group exhibited a statistically significant improvement in handgrip strength after 10 treatment sessions compared with baseline, whereas the control group showed no significant change. The increase in handgrip strength over time in the intervention group, along with the notable upward trend in the group-level linear regression analysis, suggests that the effect of hand massage may extend beyond pre- to post-treatment differences, potentially leading to progressive improvement over time. The significant increase observed in about half of the patients in individual regression analyses suggests that the efficacy of massage therapy may vary among individuals, with patient-specific clinical variables potentially influencing treatment response. The improvement in handgrip strength is may be associated with soft-tissue relaxation, enhanced circulation, reduced muscle spasm, and altered pain perception [8, 9]. Consistent with our findings, Field et al. [23] documented a notable increase in grip strength and a reduction in pain at the end of the treatment session in the group that underwent moderate-pressure massage. The findings indicate that hand massage might serve as a supplementary option for maintaining functional capacity and enhancing strength in early-stage RA patients without deformities.

The findings support the beneficial impact of massage therapy on pinch strength. This outcome suggests that hand massage may have beneficial effects, particularly on the activation of intrinsic hand muscles and on the coordination of the thumb and index finger, which are critically important for fine motor skills [24]. The absence of a significant difference in pinch strength between the groups at the end of therapy suggests that the two-week assessment period may have prevented the identification of a meaningful difference. Moreover, because pinch strength engages more specific muscle groups and requires finer motor control than handgrip strength, it is anticipated that greater benefits may be observed with prolonged treatment. Consequently, research with larger sample sizes and longer follow-up periods is required to more definitively illustrate the impact of hand massage on pinch strength.

No significant change in dolorimeter-assessed pain threshold was observed in the group that received hand massage. Likewise, no intra-group change in pain threshold was detected in the control group. This study indicates that short-term hand massage does not significantly affect the pressure pain threshold in patients with RA at an early stage of disease and with low disease activity or remission. While the pain-relieving effects of massage therapy have been documented [25, 26], the stability of the pain threshold as an objective measure, which exhibits a more subdued response to short-term interventions, may account for this observation. The patients’ lower baseline pain levels in the study, attributable to the absence of active hand-wrist arthritis and low disease activity, may have limited the assessment of the impact of massage therapy on pain threshold.

No significant difference in tactile sensory threshold, measured by the SWMT, was observed between pre- and post-treatment assessments in the intervention and control groups. These data suggest that the short-term hand massage approach used in early-stage, low-disease-activity/remission RA patients does not significantly affect superficial sensation. Moreover, the intervention may have underprioritized sensory retraining, and the total duration of 10 sessions may have been insufficient for sensory adaptation and the onset of neurosensory modifications [27]. The preservation of sensory function in the initially selected patients, attributable to the absence of active hand-wrist arthritis and considerable structural damage, may account for this outcome.

A plausible explanation for the enhancement in grip strength and hand function, despite no alterations in pain or tactile sensory thresholds, might be related to the mechanisms addressed by the implemented massage approach. The structured intervention predominantly emphasized soft-tissue relaxation, increased circulation, and periarticular movement, rather than targeted neurosensory modulation. The mechanical and circulatory effects may have enhanced muscle performance and decreased periarticular stiffness, therefore increasing functional outcomes without modifying objective sensory thresholds. The brief duration of the intervention and follow-up may have been insufficient to elicit detectable changes in neurosensory measures, which typically require prolonged stimulation and adaptation. Moreover, because the included patients had low disease activity and lacked active hand arthritis, baseline sensory impairment may have been minimal, thereby limiting the ability to detect substantial alterations in pain and touch thresholds.

The substantial decrease in DHI scores at the end of the massage therapy suggests that hand massage is beneficial for functional limitations in daily activities. This enhancement is clinically significant because the DHI specifically evaluates self-care and domestic activities that require hand dexterity [28]. This finding suggests that massage may enhance hand function by promoting soft-tissue relaxation, reducing periarticular stiffness, and improving range of motion.

The significant reduction in PGA scores within the intervention group at the end of the therapy program indicates that hand massage may influence patients’ overall disease perception and subjective well-being. The absence of a notable change in PGA in the control group, along with the observed benefit in the intervention group, suggests that this improvement may be attributable to massage therapy rather than standard follow-up procedures.

This study has some limitations. The small sample size diminishes the generalizability of the findings. The absence of blinding in the study may have increased the risk of bias. Moreover, the restricted two-week follow-up period precluded assessment of the long-term benefits and sustainability of hand massage. The restriction to early-stage, seropositive, low-disease-activity or remission female patients without radiological hand damage constrains the generalizability of the findings to patients with varying disease activity levels, advanced-stage RA, and male patients. Gender-related differences in muscle strength, pain perception, and response to massage therapy may affect outcomes.

## Conclusion

The interest in complementary approaches to the management of autoimmune rheumatic diseases, including massage therapy, has been steadily increasing [29]. This study demonstrated that a 10-session hand massage program administered to early-stage, seropositive RA patients without radiographic hand damage and with low disease activity or remission may enhance hand grip strength and hand function. Nonetheless, no substantial alterations were noted in pain and tactile sensory thresholds. The absence of flare-ups, adverse effects, and patient dropouts during the application period suggests that hand massage is well tolerated in this patient group and can be a safe complementary intervention. This pilot study suggests that a structured hand massage protocol, a low-risk method, can be integrated into rehabilitation programs to prevent early functional decline. 

## Supplementary Information

Below is the link to the electronic supplementary material.

Supplementary Material 1

Supplementary Material 2

Supplementary Material 3

Supplementary Material 4

Supplementary Material 5

Supplementary Material 6

Supplementary Material 7

Supplementary Material 8

Supplementary Material 9

Supplementary Material 10

Supplementary Material 11

## Data Availability

Raw data are available upon reasonable request from the corresponding author.

## Declarations

### Open Access Publishing

After the acceptance of the manuscript, an application will be submitted to TÜBİTAK (The Scientific and Technological Research Council of Türkiye) for open-access publishing support.
